# Melatonin in Youth: N-of-1 trials in a stimulant-treated ADHD Population (MYNAP): study protocol for a randomized controlled trial

**DOI:** 10.1186/s13063-016-1499-6

**Published:** 2016-07-29

**Authors:** Salima Punja, Catherine J. Nikles, Hugh Senior, Geoffrey Mitchell, Christopher H. Schmid, Helen Heussler, Manisha Witmans, Sunita Vohra

**Affiliations:** 1 Complementary and Alternative Research Education (CARE) Program, Department of Pediatrics, University of Alberta, #1702 College Plaza, 8512-112 Street NW, Edmonton, Alberta T6G 2C8 Canada; 2 Center for Clinical Research, The University of Queensland, Brisbane, Queensland Australia; 3 College of Health, Massey University, Auckland, New Zealand; 4 Discipline of General Practice, The University of Queensland, Brisbane, Queensland Australia; 5 Department of Biostatistics and Center for Evidence Based Medicine, School of Public Health, Brown University, Providence, Rhode Island USA; 6 Lady Cilento Children’s Hospital, Brisbane, Australia; 7 Alberta Health Services, Edmonton, Alberta Canada

**Keywords:** ADHD, Initial insomnia, Melatonin, N-of-1 trial, Randomized controlled trial

## Abstract

**Background:**

Attention-deficit/hyperactivity disorder (ADHD) is a common neurological disorder affecting 5 % of children worldwide. A prevalent problem for children with ADHD is initial insomnia. The gold standard treatment to manage ADHD symptoms is stimulant medications, which may exacerbate the severity of existing initial insomnia. Currently, no gold standard treatment option exists for initial insomnia for these children. Melatonin, a hormone and a popular natural health product, is commonly provided to children by parents and recommended by healthcare providers, but high quality pediatric evidence is lacking.

**Methods/design:**

This trial is a multicenter randomized triple-blind, placebo-controlled, parallel-group, randomized, controlled trial (RCT), in which each participant is offered an N-of-1 trial. An N-of-1 trial is a multiple-crossover, randomized, controlled trial conducted in a single individual. For the N-of-1 trial, each participant will undergo three pairs of treatment/placebo periods; each period is 1 week in length. Half the participants will have melatonin in the first period, the other half will start with placebo, and this will make up the parallel-group RCT. The primary outcome will be mean difference in sleep onset latency as measured by sleep diaries. A comparison of treatment effects yielded by the RCT data versus the aggregated N-of-1 trial data will also be assessed.

**Discussion:**

This trial will provide rigorous evidence for the effectiveness of melatonin in children with ADHD on stimulants who experience initial insomnia. Further, this study will provide the first prospectively planned head-to-head comparison of RCT data with pooled data from a series of N-of-1 trials. Aggregated N-of-1 trials may be a powerful tool to produce high quality clinical trial evidence.

**Trial registration numbers:**

ClinicalTrials.gov, NCT02333149. Registered on 16 December 2014. Australian New Zealand Clinical Trials Registry, ACTRN12614000542695. Registered on 21 May 2014.

**Electronic supplementary material:**

The online version of this article (doi:10.1186/s13063-016-1499-6) contains supplementary material, which is available to authorized users.

## Background

Attention-deficit/hyperactivity disorder (ADHD) is the most common neurodevelopmental disorder in children [1, 2] affecting 5–13 % of Canadian, Australian, and American school-aged children [3, 4]. ADHD is characterized by inattention, hyperactivity, and impulsivity, which negatively interfere with the child’s socialization and education. It has long-term ramifications throughout adulthood [5], leading to an academic underachievement, language and literacy problems, fewer years of completed schooling, lower-ranking employment, and lower achieved social class than control groups [6–11]. The utilization and cost of healthcare services for children with ADHD in the United States are approximately double those for children without ADHD. Pelham and colleagues reported that in 2005, the societal cost of childhood ADHD was approximately $42.5 billion USD [12, 13].

The first line of treatment for ADHD is stimulant medication, which is effective in 70–90 % of school-aged children [14–16]. The most commonly prescribed stimulants, methylphenidate, and amphetamines experienced a fivefold increase in use from 1987 to 2002 among children in the United States [17]. Similarly, between 1990 and 2000, Australia saw a fivefold increase in the number of children and adolescents with ADHD who had commenced taking stimulant medication [18]. Although stimulants help to effectively manage ADHD symptoms, they are also associated with a number of adverse events: initial insomnia is among the most commonly reported, and is characterized by prolonged sleep onset latency (SOL).

In general, sleep problems are among the most common complaints in childhood, with a prevalence of 6 %, as reported by parents [19]. In comparison, 50–60 % of children with ADHD experience sleep problems [20]; when children with ADHD are treated with stimulant medication, the prevalence of initial insomnia increases to 64–70 % [21]. Sleep problems not only have negative impacts on a child’s social, physical, and mental well-being [20], but lead to exhaustion and high stress levels in parents [22].

### Treatment of insomnia

Nonpharmacological interventions such as improving sleep hygiene are generally preferred as first-line treatment. Sleep hygiene entails a variety of strategies to promote the quantity and quality of sleep, such as avoiding strenuous play/exercise before bed; going to bed at a consistent time in a quiet, dark room; establishing consistent waking times; getting regular physical exercise; and maintaining a regular daily routine [23]. Although it is considered the first line of treatment, preliminary research suggests sleep hygiene has limited effectiveness in pediatric ADHD [24]. Weiss and colleagues found that sleep hygiene on its own only helped a small fraction (15 %) of children with ADHD [22].

The use of pharmacological agents to promote sleep is common, but evidence regarding safety and effectiveness for pediatric use is lacking [20]. Children with ADHD are more likely to be prescribed medications with hypnotic properties such as clonidine or antihistamines to promote sleep; however, the safety and effectiveness for this off-label use have not been established in children [25]. At present, no approved medications exist for childhood insomnia in Canada or Australia [26]. In the absence of approved pharmacotherapy, Canadian and Australian healthcare providers recommend the most popular natural health product (NHP) for sleep: melatonin [27].

### Melatonin

First discovered in 1958 [20], melatonin is a hormone released by the pineal gland and regulates the sleep-wake cycle [28, 29], thermoregulatory cycle [30], reproductive rhythm [31], and immune function [32]. Its secretion follows a circadian rhythm that is entrained to the light/dark cycle and is regulated by the hypothalamic suprachiasmatic nucleus (the central pacemaker of the body) [28]. The secretion of melatonin is suppressed by daylight but increases with darkness; it regulates the circadian rhythm by promoting the desire to sleep at night [28]. Individuals with neurodevelopmental disorders often have biological clock or circadian rhythm disturbances, which result in sleep disorders [29]. Children with ADHD have a delayed endogenous circadian pacemaker as measured by delayed sleep onset, dim-light melatonin onset, and time of awakening [33–35]. These findings suggest that melatonin may be particularly effective in children with ADHD to help promote sleep onset by two possible routes: as a chronobiotic and as a hypnotic.

Classified as an NHP by Health Canada (HC), melatonin is available over the counter [36]. In Australia, melatonin may be obtained with a prescription or without, by ordering it through the internet. Melatonin is widely used in pediatric populations for the treatment of sleep disorders [37]. A 2003 survey of 671 US community-based pediatricians reported that approximately 25 % of physicians recommended melatonin for sleep problems in toddlers (1–8 %) and adolescents (15–19 %) [26]. A 2013 study conducted in Australia found that melatonin was prescribed 89.1 % of the time for poor sleep initiation in children by general pediatricians [27].

Members of our team conducted a systematic review on the efficacy of melatonin for the treatment of sleep disorders in an evidence report for the Agency for Healthcare Research and Quality in 2004 [28]. We identified six trials of adults and children reporting SOL who had a secondary sleep disorder; meta-analysis favored melatonin but was nonsignificant (-13.2 min; 95 % CI: -27.3, 0.9) [38]. Our systematic review findings were limited by too few studies of inadequate sample sizes (n = 7–30). A more recent review identified four studies investigating the use of melatonin for insomnia in children [20]. These studies included an open-labeled study, two randomized controlled trials (RCTs) and one 3-year follow-up study [22, 39, 40]. All studies showed significant improvement in SOL in the treatment arms; however, the trials were limited by small sample sizes, variable SOL criteria, ADHD criteria, and treatment assessments, and a lack of generalizability.

### N-of-1 Trials

A major barrier to the conduct of RCTs of NHPs is participant access to the study product without a prescription (personal communication, National Center for CAM, US National Institutes of Health). Since melatonin is readily available in Canada, parents may (1) prefer to buy it themselves instead of risking their child being randomized to a placebo control arm or (2) contaminate an RCT by administering nonstudy melatonin to their child. An ideal alternative to an RCT is an N-of-1 trial, defined as a prospective, multiple-crossover, placebo-controlled, triple-blind, randomized trial in a single subject. In an N-of-1 trial, each participant is assured of receiving both the study medication and placebo, and thus learns whether the treatment works specifically for them or not.

Melatonin is an optimal treatment for testing within an N-of-1 trial framework for several reasons: (1) it has a short half-life (0.54–2 h) [28]; (2) no residual impact remains on the target symptom after excretion [38]; and (3) it is being used to treat an important and recurrent symptom that has a negative impact on quality of life and compliance with ADHD stimulant treatment [22].

### Why is this trial needed now?

More than 1000 controlled clinical trials have evaluated melatonin for the treatment of sleep disorders [28], but the literature for children with ADHD is limited to very few studies confounded by small sample sizes and limited clinical applicability [20]. The fact that melatonin is being widely used in this population despite the absence of high-quality clinical evidence highlights the need for a well-designed RCT. Our proposed study will overcome the recurring limitations of small sizes in pediatric trials by adequately powering the parallel-group trial and offering each participant an N-of-1 trial to promote trial enrollment. To our knowledge, this will be the first prospectively planned head-to-head comparison of parallel group RCTs and N-of-1 trials.

### Objectives

The primary objectives of this study are to (1) determine the effectiveness of weight-based dosing of melatonin for initial insomnia (i.e., reducing sleep onset latency) in children and adolescents with ADHD who are receiving stimulant medication and (2) determine whether N-of-1 trials provide similar estimates of treatment effect to parallel group RCTs.

## Methods/design

### Study design

**Fig. 1:**
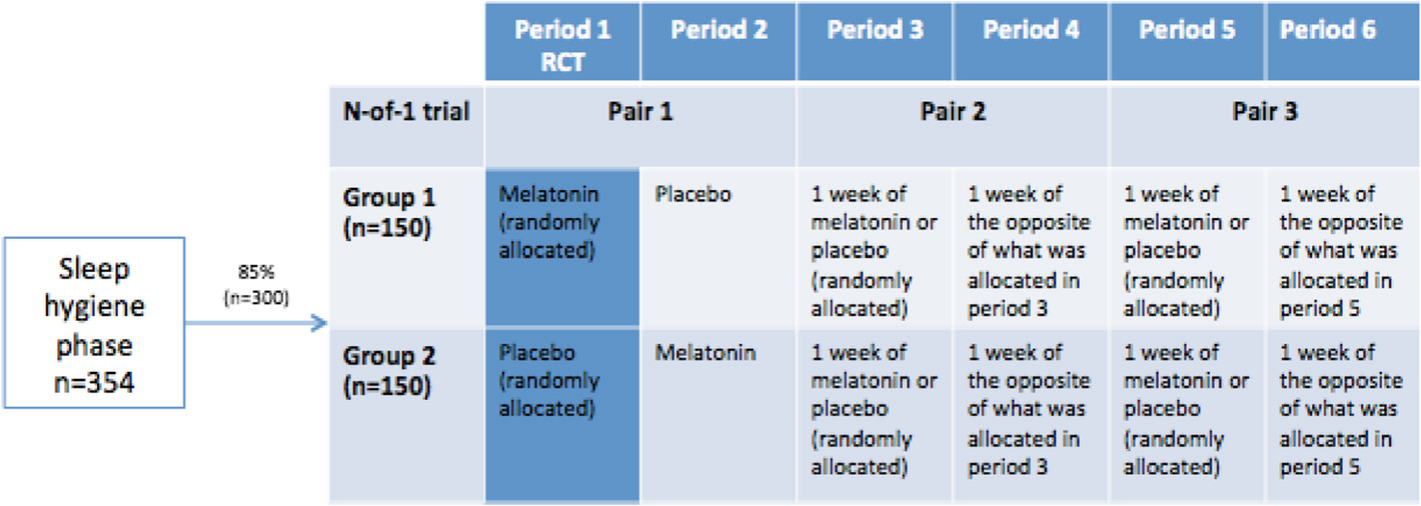
Scheme of study design

**Fig. 2:**
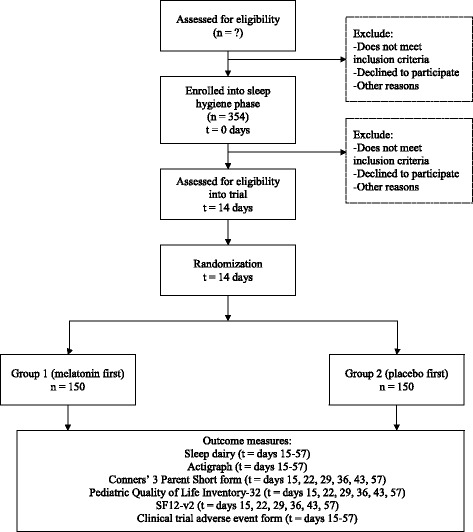
Flow of participants through study

We will conduct a prospectively planned, head-to-head comparison of a parallel-group RCT design with aggregated N-of-1 trial data. The trial is a multicenter, randomized, triple-blind, placebo-controlled series of N-of-1 trials with an embedded RCT. Half the participants will be randomly allocated to melatonin in the first period; the other half will start with placebo; and the RCT will include the data from the first period only. For the N-of-1 trial, each participant will undergo three pairs of treatment/placebo periods (each period is 1 week long, and each N-of-1 trial will last a total of 6 weeks). See Fig. 1 for scheme of the study design, Fig. 2 for flow of participants through study, and the SPIRIT checklist (Additional file 1).

### Study setting

The trial sites will be outpatient pediatric and/or family medicine clinics at hospital and community clinics in Australia and in Edmonton, Canada. Staff in all relevant clinics will be informed about the availability of the trial and will refer participants to the study investigators at each site. Community patients will also be able to be referred by their physician.

### Study participants

To be included in the trial, participants must (1) be between the ages of 6 to 17 years; (2) have a diagnosis of ADHD according to the DSM-IV criteria; (3) be on a stable dose of stimulant medication for at least 1 month prior to the study; and (4) have an SOL of ≥ 45 min, ≥ 3 nights/week, for ≥1 month. Exclusion criteria include (1) children with comorbid psychiatric/neurological diagnosis that may affect sleep; (2) children with untreated obstructive sleep apnea, untreated sleep-related breathing disorder, untreated narcolepsy, sleep-related movement disorders, parasomnias, adjustment insomnia, insomnia due to drug use or mental health issues, or secondary enuresis; (3) children with a known allergy or hypersensitivity to melatonin or other study drug ingredients; (4) children on immunosuppressive drugs, blood pressure drugs, selective serotonin reuptake inhibitors or anticoagulant drugs; and (5) children not on a regular sedative/hypnotic medication, whose parents do not agree not to alter the daily dose of these for the duration of the trial; children with active or uncontrolled disorders (hormonal disorders, diabetes, liver disease, abnormal kidney function, untreated kidney disease or blood clotting disorders); (6) children with seizure disorders; (7) breastfeeding or pregnant women; (8) female participants with child-bearing potential not practicing an acceptable form of birth control throughout the trial (in Australia, females who are ≥ 12 years who are menstruating and sexually active); (9) participants who disagree to not driving or operating heavy machinery within 8 hours of ingestion of study medication; and (10) children whose parent/primary caregiver does not understand English, have a phone, or provide informed consent.

## Interventions

### Sleep-hygiene phase

Upon recruitment, all participants/parents will receive an information sheet and a 15-min counseling session to review optimal sleep hygiene. The family will be asked to try the sleep-hygiene strategies suggested for 14 days. At day 7, the research assistant will telephone the family to ensure that sleep hygiene measures are implemented and address any barriers. If the child is still eligible for the study at day 14, the child will be offered enrollment into the trial. While sleep hygiene is an important first step, preliminary data suggest that only 15 % of children will respond [22]. Therefore, the majority of those pre-enrolled are anticipated to continue on to trial enrollment. Randomization will not occur until trial enrollment.

### Melatonin

Previous research suggests that an oral dose of 2.5–10 mg before or at the desired bedtime is well tolerated in children [41]. Similar to previous pediatric trials [40], we will use weight-based dosing, giving 3 mg of sublingual melatonin to those children who are < 40 kg and 6 mg to those ≥ 40 kg. Melatonin will be taken once daily, 30 minutes prior to bedtime. Placebo with an identical appearance has been manufactured. The clinical effect of melatonin is rapidly evident, and melatonin has a short half-life (0.54–2 h). Therefore, an appropriate duration of each treatment period is 1 week. Data from day 1 of each period will be discarded to provide a washout period. If a carryover effect is found subsequent to day 1, appropriate adjustments will be made, e.g., discarding an extra day’s data.

### Randomization

Participants will undergo three pairs of treatment with melatonin and placebo. In each pair, the order of treatment will be randomized. In particular, the first period for each patient will be randomized in variable block sizes of 4 and 6, stratified by age (<12 and ≥ 12 years) and country (Canada or Australia), to ensure a balance of the treatment groups for the parallel-group RCT. The randomization code will be developed by the study statistician. The randomization code will be provided to the central research pharmacy for each participating site and to maintain the blind. When a participant has been enrolled, the research pharmacy will randomize the subject based on the master randomization list and courier six bottles numbered 1–6, corresponding to each week’s treatment. Each bottle will contain seven capsules (or 14 for children who weigh < 40 kg) to be taken over 7 days. Randomization codes will be kept by the research pharmacy in case the code needs to be broken for safety reasons as determined by the Data Safety and Monitoring Board (DSMB).

### Blinding

The trial will be triple-blinded (participant, healthcare provider, and statistician). The placebo has the same appearance, volume, weight, odour, and taste as the active product. There will be unblinding of the individual participant’s randomized N-of-1 sequence for the healthcare provider and participant only upon receiving individual trial reports or on participant request to stop the trial early.

### Cointerventions

All participants will be encouraged to continue practicing good sleep hygiene consistently throughout their N-of-1 trial. Participants will receive other medications and healthcare tests their primary physician deems appropriate. If a child is already on the following medications, we will require stable doses of each: approved anti-depressants, anxiolytics, sedatives/hypnotics, and clonidine throughout the 6-week trial. If not already taking these medications, the child may not start them regularly during the trial. It is preferable that participants abstain from coffee, alcohol, and high caffeine energy drinks for 1 week prior to the sleep-hygiene phase, during the sleep-hygiene phase, and during the trial itself. Any caffeine intake during the previous week will be recorded during the sleep hygiene phase, at baseline, and at the end of each week during the trial.

Any changes in dose/frequency/type of stimulant treatment for ADHD and whether the patient is taking nontrial melatonin will be recorded throughout the trial.

### Contamination

Because melatonin is readily available over the counter in Canada, contamination (parents providing melatonin to their child) could potentially pose a risk. The use of N-of-1 trials is expected to prevent this from occurring, as parents are guaranteed that their child will receive melatonin during the course of the trial. To further reduce contamination, parents will be reminded that their child will be on active therapy in a blinded fashion, and they would not want to “double dose” their child. Physician information letters will be sent to the child’s doctor to remind them not to recommend melatonin during the study.

### Sample size

A proposed sample size was calculated for the parallel group RCT. The null hypothesis is that both group means are equal, and the alternative hypothesis is SOL will be 42 minutes on melatonin and 72 minutes on placebo [22]. To account for a potential dropout of 25 %, we assume that 25 % of the participants will show no effect of treatment and will therefore have a SOL of 72 minutes under both treatments. The average time in the treatment group will then be 49.5 minutes (as ¾ will have a time of 42, and ¼ a time of 72). The intent-to-treat effect will then be a 22.5-minute reduction in SOL. Assuming a pooled standard deviation of 60 minutes [22], randomization of 150 patients per group (300 total) will provide 90 % power with a type I error rate of 0.05 using a two-sided test. We will need to recruit 177 patients per group (354 total) to commence the sleep-hygiene phase in order to account for the 15 % of individuals expected to respond to sleep hygiene. Because the N-of-1 design will use multiple crossovers for each patient, this sample size will also achieve sufficient power for the aggregated N-of-1 analysis.

### Proposed trial outcome measures

#### Primary outcomes

SOL (in minutes) will be measured daily by sleep diaries completed by parents. Participants who are > 12 years will assist in filling out sleep diaries. Sleep dairies are among the most common assessment tools in sleep research [28, 42].

#### Secondary outcomes

In a subgroup of children, SOL (minutes) will also be measured daily by actigraphy, which are validated to measure sleep quality in clinical research [43]. We will use Actiwatch Spectrum (Phillips Respironics, Murrysville, PA, USA). The mean amount of time awake at night (minutes) and mean duration of sleep while in bed (minutes) will be measured daily by sleep diaries and actigraphy. The number of awakenings after sleep onset will be measured by sleep diaries. Sleep efficiency (proportion of time spent asleep while in bed) will be measured daily by actigraphs. Daytime behavior, specifically core ADHD symptoms, will be measured weekly by the Conners’ 3 Parent Short form [44]. The quality of life of the participants will be measured weekly by the Pediatric Quality of Life Inventory-32 [45]. The parent’s quality of life and fatigue will be measured weekly by the SF12-v2 [46]. See Table 1 for a list of outcome measures used in this study.

**Table 1:** List of outcome measures used

Outcome	Tool	Frequency of measurement
SOL	Sleep diary	Daily
	Actigraphy	
Amount of time awake at night	Sleep diary	Daily
	Actigraphy	
Number of awakenings after sleep onset	Sleep diary	Daily
Sleep efficiency	Actigraphy	Daily
ADHD Symptoms	Conners’ 3 Parent Short form	Weekly
Participant quality of life	Pediatric Quality of Life Inventory-32	Weekly
Parent quality of life and fatigue	SF12-v2	Weekly
Adverse events	Clinical trial adverse event form	Daily

#### Adverse events

Each participant will receive ongoing safety monitoring throughout the trial. Parents will be asked about side effects during weekly telephone follow-ups. All adverse events will be forwarded to the DSMB. The DSMB consists of three members with clinical, methodological, and NHP expertise who are independent of the trial. The DSMB will review all documented harms during the study and adjudicate them with regards to causality. Frequency and severity of any adverse events will be reported using the Clinical Trial Adverse Event form that includes open-ended questions on potential adverse reactions. Health Canada and Therapeutic Goods Australia will be informed of all serious adverse events (those warranting hospitalization) within 7 days.

### Withdrawal

If participants experience intolerable adverse events, they may choose to withdraw, or the investigators may make this decision in the best interest of the patient. Patients who are lost to follow-up, who cannot be contacted for telephone follow-ups, or who wish to discontinue medication for 7 or more consecutive days will be withdrawn. If a participant chooses to withdraw, a withdrawal report form will be completed. Assessments and reason(s) for withdrawal will be recorded.

### Early stopping (within a treatment period)

While pre-specifying the number of paired periods in an N-of-1 trial strengthens statistical analysis of the data [47], N-of-1 trials may accommodate early stopping within a treatment period. Early stopping most commonly occurs for three reasons—perceived lack of effectiveness, a dramatic improvement in the target outcome or safety concerns. If a participant/parent feels strongly that a period should be stopped early, the research nurse will give them the option of switching to the next period’s medication after day 4 of that period, rather than withdrawing from the trial. Early stopping does not compromise the validity of the data acquired as all decisions are made with the blind intact, and the randomization sequence unaltered. Early stopping within a treatment period (i.e., early progression to the next treatment period) promotes trial completion, as opposed to withdrawal, as participants recognize that we will try to accommodate their requests within the confines of the study.

### Statistical analysis plan

We will undertake (1) a standard parallel-group RCT analysis comparing results from the first period for treatment and control groups; (2) analysis of individual N-of-1 trials; and (3) aggregated analysis across N-of-1 studies using data from all periods in all participants. Each analysis will report an estimate of the mean difference between treatment groups across the measurements taken over the course of the trial together with 95 % probability intervals and a probability estimate of the likelihood of a treatment effect. Primary analyses will use the Bayesian method with noninformative prior distributions. We will also carry out analyses based on reasonable weakly informative priors and on the likelihood alone as a check on sensitivity to assumptions about the prior distribution. Data on randomized patients at baseline will be summarized with descriptive statistics (mean, median, standard deviation, and ranges) for quantitative variables, and counts and percentages for categorical variables. Regression adjustments will be carried out for variables found to be unbalanced at baseline between treatment and placebo groups. A variety of interaction tests will be carried out to test for subgroup differences.

#### Parallel-group RCT analysis

Using data from the first treatment period, we will compare those who received melatonin with those who received placebo based on intent-to-treat for all outcomes adjusted for the average baseline SOL determined by the pre-randomization sleep-hygiene phase. Outcomes measured daily will be analyzed both with time series methods and by using the outcome averaged across the treatment period. Outcomes measured weekly provide only one measurement per participant. Secondary analyses will examine effects defining treatment as that actually received.

#### Analysis of individual N-of-1 trials

At the end of each participant’s N-of-1 trial, a patient report will be forwarded to the referring physician in order to guide treatment decision. The patient report will present (1) the child’s SOL (according to parent diaries) for each day of the study; (2) the child’s mean and median SOL on both treatments; (3) whether the child’s SOL has normalized below 30 minutes; (4) whether the SOL is within the parent’s expectations of significant improvement; and (5) any reported adverse events. The patient and physician will make an individual decision about the continued use of melatonin.

#### Aggregated analysis

N-of-1 trials will be combined using a multilevel random effects model that incorporates variance within and between patients. This within-patient level will incorporate variation resulting from the treatment crossovers as well as time effects modeled in various forms (e.g., linear trends, nonlinear trends, and carryover effects). Each within-patient regression coefficient will be treated as a random effect that can vary based on these between-patient factors. The primary analysis will calculate the average treatment effect (i.e., will model the mean of the individual treatment effects as a constant). Secondary analyses will model the mean as a function of factors that vary among patients such as age, sex, and dose. These terms describe the main effects of these factors, their interactions with the treatment, and their interaction with time. A full list of the factors evaluated for these subgroup analyses is given below.

The full multilevel model will return estimates of both average effects across the population of patients, as well as effects for individuals informed by the results on other participants. The predicted effects for an individual are weighted averages of the individual’s data and the averages from others. This model incorporates correlations among the measurements within an individual. It also enables comparison of the individual’s predicted treatment effect using the multilevel model with that from using only that individual’s data.

Carryover, the phenomenon whereby the effects of one treatment may continue to affect the outcomes measured after the next treatment is started, is always a risk in crossover studies. It is not expected that placebo effects would carry over into a melatonin period, but melatonin effects may carry over into a placebo period. Carryover might also differentially affect measurements made in a melatonin treatment period that follows another melatonin treatment period. We will investigate various adjustments for carryover, such as discarding the first measurements in a treatment period on outcomes measured daily, to determine whether results are sensitive to carryover. These adjustments will allow for potential differential carryover by treatment regimen.

Bayesian models will be estimated using Markov chain Monte Carlo with model assessment using posterior predictive checks [48] and the deviance information criterion [49]. Non-Bayesian models will be fit using generalized linear and nonlinear mixed models.

#### Missing data

For the aggregated N-of-1 trial analysis, we will develop appropriate missing data models based on the data-missingness mechanism. We will first check if the data are missing completely at random and if not, as is likely, will adjust for observed variables related to missingness using, if possible, longitudinal fixed or random effects models, assuming the mean and covariance structures are known. If considerable data are missing on key predictors, we will use multiple imputation. As a sensitivity analysis, we will also analyze differences between groups at each time point imputing a change of zero for those missing follow-up.

#### Interim safety analysis

After 100 participants have been enrolled or after one year, whichever occurs first, the DSMB will supervise one interim analysis for safety only using treatment labels (A and B) without knowledge of the identity of each label to determine if there is an excess of adverse events in one group. A *p* value of 0.10 will be used as a decision criterion for stopping the study for harm. As no effectiveness endpoint will be evaluated, there will be no need to adjust the sample size.

#### Subgroup analyses

Primary subgroup analyses include the two main stratification factors: (1) age (<12, ≥ 12 years) and (2) country (Australia and Canada). Exploratory analyses will look at (3) the type of stimulant, (4) weight-adjusted dose of stimulant, (5) presence of psychiatric comorbid illness, (6) gender, (7) calendar time of enrollment as a proxy for differences by climactic season, (8) severity of SOL, and (9) referring physician/clinic.

### Data management

The Women and Children’s Health Research Institute (WCHRI) at the University of Alberta will be in charge of data management. WCHRI staff will check data entered online by participants/parent/guardians and research assistants, identifying queries for research assistants to resolve. Each participant’s N-of-1 data will be communicated back to each participant’s physician by fax directly, thereby fulfilling our obligation to all participants without biasing staff (i.e., project manager, research assistants, and study statistician will not be informed of the trial results as they accrue). Regular data quality checks will be undertaken through automated data validation checks. Data collected from the actigraphs will be downloaded to a computer and reviewed for completeness and errors.

## Discussion

### Impact of research

A great need exists to address the current information gap regarding the effectiveness of melatonin use on sleep disturbances in children with the greatest clinical need such as those on stimulants to treat ADHD. Our novel approach will not only answer this clinical question, it will also allow a rigorous comparison of the advantages and disadvantages of aggregated N-of-1 trials versus a parallel-group RCT. This method has the potential to become the gold standard in the accumulation of high-grade evidence to support clinical therapies in chronic disease management.

### Trial governance

The study will be overseen by the Trial steering committee (TSM), which is comprised of the Principal investigator, co-investigators, research coordinator, and study statistician. The TSM will be responsible for ensuring that the study is conducted within appropriate and professional ethical guidelines, ensuring the good clinical practice guidelines are observed at all times. Furthermore, the DSMB, which consists of three members with clinical, methodological, and NHP expertise who are independent of the trial will review all documented harms during the study and adjudicate them with regard to causality.

Any significant amendments to the protocol will be submitted to Health Canada, Therapeutic Goods Australia, and to the University of Alberta and University of Queensland ethics committees for approval. Trial registries will also be notified of any major amendments.

The trial sponsor is the University of Alberta. The sponsor has no role in the trial design; collection, management, analysis or interpretation of data; writing of reports; and submission for publication.

### Trial status

Recruitment began in March 2015.

## Abbreviations

ADHD, attention deficit/hyperactivity disorder; DSMB, Data Safety And Monitoring Board; NHP, natural health product; RCT, randomized controlled trial; SOL, sleep onset latency; TSM, trial steering committee; WCHRI, Women and Children’s Health Research Institute

## Additional file

Additional file 1: SPIRIT 2013 Checklist: Recommended items to address in a clinical trial protocol and related documents. (DOCX 61 kb)
